# The effect of an interactive delirium e-learning tool on healthcare workers’ delirium recognition, knowledge and strain in caring for delirious patients: a pilot pre-test/post-test study

**DOI:** 10.1186/s12909-016-0537-0

**Published:** 2016-01-15

**Authors:** Elke Detroyer, Fabienne Dobbels, Deborah Debonnaire, Kate Irving, Andrew Teodorczuk, Donna M. Fick, Etienne Joosten, Koen Milisen

**Affiliations:** Department of Public Health and Primary Care, Academic Centre for Nursing and Midwifery, KU Leuven, Kapucijnenvoer 35 - PB 7001/4, 3000 Leuven, Belgium; Group Health and Welfare, UC Leuven-Limburg, Genk, Belgium; Department of Nursing, Dublin City University, Dublin, Ireland; School of Medical Education, Newcastle University, Newcastle upon Tyne, UK; Campus for Ageing and Vitality, Newcastle upon Tyne, Northumberland Tyne and Wear NHS Foundation Trust, Newcastle upon Tyne, UK; College of Nursing, The Pennsylvania State University, University Park, Pennsylvania, USA; Department of Psychiatry, College of Medicine, The Pennsylvania State University, University Park, Pennsylvania, USA; Department of Geriatrics, University Hospitals Leuven, Leuven, Belgium

**Keywords:** Delirium, Education, E-learning

## Abstract

**Background:**

Studies investigating the effectiveness of delirium e-learning tools in clinical practice are scarce. The aim of this study is to determine the effect of a delirium e-learning tool on healthcare workers’ delirium recognition, delirium knowledge and care strain in delirium.

**Methods:**

A pilot pre-posttest study in a convenience sample of 59 healthcare workers recruited from medical, surgical, geronto-psychiatric and rehabilitation units of a university hospital. The intervention consisted of a live information session on how to use the e-learning tool and, a 2-month self-active learning program. The tool included 11 e-modules integrating knowledge and skill development in prevention, detection and management of delirium. Case vignettes, the Delirium Knowledge Questionnaire, and the Strain of Care for Delirium Index were used to measure delirium recognition, delirium knowledge and experienced care strain in delirium respectively. Subgroup analyses were performed for healthcare workers completing 0 to 6 versus 7 to 11 modules.

**Results:**

The delirium recognition score improved significantly (mean 3.1 ± SD 0.9 versus 2.7 ± 1.1; *P* = 0.04), and more healthcare workers identified hypoactive (*P* = 0.04) and hyperactive (*P* = 0.007) delirium in the posttest compared to the pretest phase. A significant difference in the change of recognition levels over time between the 0 to 6 and 7 to 11 module groups was demonstrated (*P* = 0.03), with an improved recognition level in the posttest phase within the 7 to 11 module group (*P* = 0.007). After adjustment for potential confounders, this difference in the change over time was not significant (*P* = 0.07) and no change in recognition levels within the 7 to 11 module group was noted (*P* = 0.19). The knowledge score significantly improved in the posttest compared to the pretest phase (mean 31.7 ± SD2.6 versus 28.3 ± 4.5; *P* < 0.001), with a significant increased level within the 7 to 11 module group (unadjusted *P* < 0.001/adjusted *P* = 0.02). Overall, no difference between posttest and pretest phases was documented for care strain (*P* = 0.46).

**Conclusion:**

The e-learning tool improved healthcare workers’ delirium recognition and knowledge. The effect of the tool is related to its level of completion, but was less explicit after controlling for potential confounders and warrants further investigation. The level of strain did not improve.

## Background

Delirium is a common disorder in older hospitalized patients, characterized by an acute and fluctuating disturbance in attention and awareness; and a disturbance in cognition or perception [1, 2]. Although delirium is potentially preventable and treatable, healthcare workers often lack the necessary knowledge, attitudes or skills to address risk factors systematically and detect or manage delirium cases effectively [3–6], which might adversely affect patient outcomes and increase clinicians’ workload [2, 7].

Educational strategies including reinforcing (i.e., use of reminders and feedback from experts) and enabling (i.e., use of guidelines, pocket cards or protocols) approaches have shown to be effective in improving delirium care, with benefits on the incidence, duration and severity of delirium, functional status and length of stay as well as on healthcare workers’ knowledge, skills and workload [8–10]. However, implementing and maintaining adherence to these multifactorial educational initiatives is time consuming and labour intensive, and thus these initiatives are difficult to implement outside the research setting [11, 12]. Furthermore, given the variety of healthcare workers involved in the care for delirious patients, broader approaches to education targeting the mixed learning needs of the whole multidisciplinary team are needed [11].

E-learning has been described as a novel approach that facilitates delivery of education for large groups of people as well as providing a more flexible and cost-effective method of training [11, 13, 14]. It can be defined as “learning facilitated and supported through the use of information and communication technology that can cover a spectrum of activities from the use of technology to support learning as part of a ‘blended’ approach, to learning that is delivered entirely online. Whatever the technology, learning is the vital element” [15].

A systematic review showed that e-learning improves knowledge, skills and behaviours of healthcare workers across different healthcare domains [16]. Despite its relevance, studies investigating the effectiveness of delirium e-learning tools in clinical practice are scarce. To our knowledge, only two studies have evaluated the use of delirium e-learning on nursing outcomes and revealed positive effects on delirium recognition and knowledge [17, 18]. However, some critical information was lacking regarding the instrument used to measure delirium knowledge [17], the specific content of the intervention [18], or compliance with using the e-learning tool [17, 18]. Moreover, those studies did not focus on other nursing outcomes (e.g., attitudes, documentation of delirium in nursing records, levels of strain when caring for patients with delirium). A descriptive study highlighted an association between delirium training and lower levels of strain of care [19], yet no study investigated whether healthcare workers’ level of burden when caring for delirious patients might be sensitive to delirium e-learning education also.

The aim of this intervention study was to determine the effect of a delirium e-learning tool on healthcare workers’ knowledge about delirium, their ability to recognize delirium and subjective strain experienced when caring for patients with delirium.

## Methods

### Design, setting and population

A pilot pre-test/post-test study was conducted in a convenience voluntary sample of healthcare workers (except for 2 physiotherapists and 2 occupational therapists being staff members of the participating units, all of them were nurses) recruited from 20 adult inpatient units of a university hospital. The units (e.g., medical, surgical, geronto-psychiatric and rehabilitation units) were selected based on their head nurses’ willingness to participate. All healthcare workers working on the participating units were eligible for inclusion.

### Intervention

The intervention included the use of an on-line self-directed delirium educational tool for healthcare workers, which integrates knowledge and skill development in delirium prevention, detection and management. This e-learning tool was developed by the research team and is freely accessible in Dutch language at www.deliriummodule.be. More details about the development and feasibility testing have been reported previously [11, 20].

The e-learning tool is organized in 11 modules, and provides a wide range of information about delirium specifics (occurrence rates, clinical presentation, types, risk factors, experiences of patients), delirium prevention and treatment strategies, and information about the use of screening instruments for delirium detection (Table 1). It takes between 5 and 15 min to complete one module. The estimated time to complete the entire tool is 2 to 2.5 h. To achieve a deeper understanding of delirium with integration of acquired items into practice, theory is combined with videos (e.g., examples of hypoactive and hyperactive delirium performed by actors, the use of screening instruments), case studies and tests for self-assessment composed of multiple answers (2 of more possibilities but only one answer is correct) with feedback.

**Table 1:** Overview of the Different Modules Within the Delirium E-learning Tool

Module	Themes
1 Occurrence and consequences	❖ Introduction ❖ Occurrence ❖ Consequences
2 Clinical presentation	❖ Introduction ❖ Features of delirium ❖ Motoric subtypes
3 Exercises in delirium recognition	❖ Introduction ❖ Exercises in delirium recognition
4 Differences between delirium, dementia and depression	❖ Introduction ❖ Differentiation between the three D’s ❖ Exercises
5 Predisposing and precipitating risk factors	❖ Introduction ❖ Multifactorial risk model ❖ Exercises
6 Screening for delirium	❖ Introduction ❖ Screening instruments - Delirium Observation Screening Scale and its use (video) - Confusion Assessment Method (CAM) – CAM-ICU o Mini-Mental State Examination and its use (video) o Attention tests and its use (video) ❖ Systematic screening ❖ Exercises
7 Prevention of delirium	❖ Introduction ❖ Screening patients at risk & prevention strategies ❖ Early detection
8 Treatment of delirium	❖ Introduction ❖ Identification causes ❖ Treatment of delirium caused by alcohol or benzodiazepines withdrawal ❖ Treatment of delirium caused by other factors ❖ How to deal with aggressive patients
9 Family and relatives	❖ Introduction ❖ Experiences family members/how to support ❖ Experiences patients/how to support
10 Overall roadmap/algorithm	❖ Introduction ❖ Flowchart management risk patients and management delirium
11 Case study ‘Ants in the tea”	❖ Introduction ❖ Case study ‘Ants in the tea’ - Case history - Patient anamnesis/ delirium detection in the hospital - Family anamnesis - Identification of causes - Treatment - Evaluation

The intervention started with a one-hour live information session to deliver participants a personal log-in code and to provide them with oral and written information about using the e-learning tool. Subsequently, the tool was available for 2 months during which participants were asked to access the delirium course at least once. Because the tool was based on self-active learning, participants could start, finish and re-start at any time. After 1 month, all participants received an e-mail reminder to encourage completion of the educational tool.

### Variables and measurements

Data were collected at 2 time points during the study between December 2010 and May 2011, immediately before the educational intervention and after the 2-month learning period. Baseline and follow-up data were measured using four questionnaires, including a questionnaire for demographic information and three questionnaires to assess (1) delirium recognition as primary outcome, and (2) knowledge about delirium and (3) experienced strain in caring for patients with delirium as secondary outcomes.

#### Demographic and professional data

The following data were collected: age, gender, number of years of work experience, employment status, day- or night work, level of education and education in delirium attended during the last 5 years before the start of the study.

#### Delirium recognition

The ability to identify delirium was measured with standardized ‘cases vignettes’ [21]. These validated vignettes contain five different cases about hospitalized patients with dementia, hypoactive delirium, hyperactive delirium, hypoactive delirium superimposed on dementia (DSD) or hyperactive DSD. Four of them were used in the pretest phase (i.e., dementia, hypoactive delirium, hyperactive delirium and hyperactive DSD). In the posttest phase, the hyperactive DSD case was replaced by the case with the hypoactive DSD patient. For each case, of which all had one single correct answer, the patient’s mental status had to be scored as having dementia, delirium, delirium superimposed on dementia, normal ageing, depression or none of the options. Total delirium recognition (DR) score is the sum of the correct answers, and ranges from 0 to 4.

#### Knowledge about delirium

A 35-item true-false Delirium Knowledge Questionnaire (DKQ), which includes 23 of the 28 items from the ‘knowledge’ questionnaire of Hare et al. [22], was developed by the research team to assess knowledge about delirium classified into three relevant domains: 1) knowledge related to the presentation, symptoms and outcomes of delirium (*n* = 10 items), 2) its causes and risk factors (*n* = 11 items), and 3) delirium prevention and management strategies (*n* = 14 items) (Table 2). Total DKQ score is the sum of the correct answers and ranges between 0 and 35. Because no existing questionnaire measures all of these knowledge domains, the DKQ was developed. It was based on the questionnaire of Hare et al. [22], which focuses on two knowledge domains: 1) delirium presentation, symptoms and outcomes, and 2) risk factors and causes. Questionnaire development comprised different steps. First, items were reproduced (items 1–10, 12–14, 16, 19–22), modified (items 11, 15, 17, 18, 23), or generated to measure all relevant aspects of knowledge about 1) delirium presentation, symptoms and outcomes, 2) its risk factors and causes, and 3) its prevention and management strategies. Second, the content of the newly developed Delirium Knowledge Questionnaire was evaluated by an independent multidisciplinary panel of experts (e.g., one geriatrician, one psychologist, three researchers with nursing background and two nurses), and face validity was tested in 4 nurses.

**Table 2:** Proportion of Correct Answers on the Delirium Knowledge Questionnaire in Healthcare Workers in the Pretest and Posttest Phase (*n* = 59)

Items	Pretest phase (*n* = 59)	Posttest phase (*n* = 59)
Items related to knowledge about the presentation, symptoms and outcomes of delirium, n correct (%)		
1. Fluctuation between orientation and disorientation is a typical feature of delirium	40 (67.8)	46 (78)
2. Symptoms of depression may mimic delirium	47 (79.7)	54 (91.5)
3. Patients never remember episodes of delirium	41 (69.5)	52 (88.1)
4. Delirium never lasts for more than a few hours	53 (89.8)	57 (96.6)
5. A patient who is lethargic and difficult to rouse does certainly not have a delirium	51 (86.4)	55 (93.2)
6. Patients with delirium are always physically and/or verbally aggressive	49 (83.1)	55 (93.2)
7. Patients with delirium have a higher mortality rate	35 (59.3)	50 (84.7)
8. Behavioral changes in the course of the day are typical of delirium	48 (81.4)	55 (93.2)
9. A patient with delirium is likely to be easily distracted and/or have difficulty following a conversation	53 (89.8)	58 (98.3)
10. Patients with delirium will often experience perceptual disturbances (e.g., visual and/or auditory hallucinations)	58 (98.3)	59 (100)
Items related to knowledge about causes and risk factors of delirium		
11. A patient admitted with pneumonia and having diabetes, visual and auditory disturbances has the same risk for delirium as a patient admitted with pneumonia without co-morbidities	31 (52.5)	44 (74.6)
12. The risk for delirium increases with age	47 (79.7)	51 (86.4)
13. A patient with impaired vision is at increased risk of delirium	36 (61.0)	55 (93.2)
14. The greater the number of medications a patient is taking, the greater their risk of delirium	31 (52.5)	41 (69.5)
15. A urinary catheter reduces the risk of delirium	49 (83.1)	49 (83.1)
16. Poor nutrition increases the risk of delirium	48 (81.4)	59 (100)
17. Dementia is an important risk factor for delirium	45 (76.3)	48 (81.4)
18. Diabetes is an important risk factor for delirium	37 (62.7)	21 (35.6)
19. Dehydration can be a risk factor for delirium	56 (94.9)	59 (100)
20. Delirium is generally caused by alcohol withdrawal	56 (94.9)	56 (94.9)
21. A family history of dementia predisposes a patient to delirium	44 (74.6)	47 (81.0)
Items related to knowledge about delirium prevention and management strategies		
22. Treatment of delirium always includes sedation	49 (83.1)	54 (91.5)
23. Daily use of the Mini-Mental State Examination (MMSE) is the best way for diagnosing delirium	36 (61.0)	35 (59.3)
24. Providing as much staff as possible to take care at the patients’ bedside is an important strategy in the prevention of delirium	59 (100)	59 (100)
25. The use of physical restraints in patients at risk for delirium is the best way to ensure their safety	53 (59.8)	56 (94.9)
26. Encouraging patients to (correctly) wear their visual/hearing aids is necessary to prevent delirium	46 (78.0)	59 (100)
27. Adequate hydration is an important strategy in the prevention of delirium	55 (93.2)	59 (100)
28. The maintenance of a normal sleep-wake cycle (e.g., avoidance of sleep interruption) is an important strategy in the prevention of delirium	55 (93.2)	58 (98.3)
29. The use of haloperidol in preoperative surgical fracture patients is a way to prevent delirium	54 (91.5)	51 (86.4)
30. The stimulation of patients to perform different activities at the same time is a way to prevent delirium	59 (100)	58 (98.3)
31. Keeping instructions for patients as simple as possible is important in the prevention of delirium	50 (84.7)	52 (88.1)
32. Early activation/ambulation (e.g., getting patients out of bed as soon as possible) of patients is an important strategy in the prevention of delirium	40 (67.8)	55 (93.2)
33. Providing patients with familiar objects (e.g., photos, clock, newspaper) is important to prevent sensory deprivation	48 (81.4)	55 (93.2)
34. Avoid eye contact in the prevention of delirium because it can be seen as a threat	59 (100)	57 (96.6)
35. Keeping oral contact with the patient is an important strategy in the prevention of delirium	46 (78)	53 (89.8)

#### Strain in caring for delirious patients

Subjective strain in caring for delirious patients was measured with the Strain of Care for Delirium Index (SCDI) [23]. This scale contains 20 characteristics of delirious behavior, presented within four subscales: hypoactive behavior (*n* = 3 items), hypoalert behavior (*n* = 4 items), fluctuating course and psychoneurotic behavior (*n* = 5 items), and hyperactive/hyperalert behavior (*n* = 8 items). The items are scored on a four-point Likert scale ranging from ‘quite easy to cope with’ (score 1) to ‘quite difficult to cope with’ (score 4). Total scores range between 20 and 80, with higher scores indicating greater difficulty in coping with delirious behaviors.

#### Completion of e-learning tool and time to complete

The number of modules completed by each healthcare worker was registered, and ranges from 0 to 11. Furthermore, healthcare workers were asked to give times to complete the e-learning tool.

### Ethics

The study was approved by the Medical Ethics Committee of the Leuven University Hospitals.

### Analysis

Only healthcare workers who did not complete the post-test questionnaires were excluded. Descriptive analysis were performed to examine demographic and professional data, and to summarize the results of the ‘Case Vignettes’, the Delirium Knowledge Questionnaire (DKQ) and the Strain of Care for Delirium Index (SCDI). Categorical data were expressed as absolute numbers and percentages; continuous data as means and standard deviations. Data from the ‘Case Vignettes’ and DKQ were not only analyzed at participant level (e.g., total delirium recognition (DR) score and total DKQ score, respectively), but also at case/item level. At this level, answers were classified as ‘correct’ or ‘incorrect’ (e.g., each case/item had a single correct answer) and proportions of correct cases/items were calculated.

First, differences in scores between the pre-test and post-test phase were analyzed for participants who completed at least one e-learning module. McNemar’s tests were used to test differences in proportions of correct answers on the four ‘Case Vignettes’ separately. Differences in total DR scores, total DKQ scores, total SCDI scores and SCDI subscale scores were evaluated using paired t-tests for normally distributed data and the Wilcoxon Signed Rank test for non-normally distributed data. Effect sizes were calculated using Cohen’s d and expressed as small (0.2–0.5), moderate (0.5–0.8), or large (>0.8) differences [24].

Second, all participants who completed pre- and posttest questionnaires were included in the analysis. They were further categorized into two a prior subgroups: low/moderate completion subgroup (0–6 modules); good/excellent completion subgroup (7–11 modules). To examine changes in outcome variables (e.g., level of recognition, level of knowledge, level of strain of care) between these subgroups over time, three linear mixed models for repeated measures were built. Per model, the outcome measurements were included (model 1: DR scores; model 2: DKQ scores; model 3: SCDI scores), with subgroup, time point (T1 pretest phase, T2 posttest phase) and their interaction as explanatory variables. To correct for confounding factors, two potential confounders were included in the analysis: number of years of work experience, and employment status. Because of the high correlation between ‘number of years of work experience’ and ‘age’ (*r* = 0.93), the variable age was not included in the model.

The association between the number of completed e-learning modules and the change scores (e.g., change in post – pretest scores) of the total DR scores, total DKQ scores and total SCDI scores were calculated with the Pearson’s r or Spearman’s rho correlation coefficient depending on the distribution of the data.

Data were analyzed using SPSS version 16 (SPSS, Inc., Chicago, IL) and SAS version 9.2 (SAS Institute Inc., Cary, NC). Statistical significance was set at *P* < 0 .05 and all tests were two-sided.

## Results

### Sample

Seventy-two healthcare workers agreed to participate, of whom 13 were excluded because they only completed the pretest. Characteristics of the 59 included healthcare workers are shown in Table 3. No differences were observed between excluded and participating healthcare workers.

**Table 3:** Characteristics of the Healthcare Workers (*n* = 59)

	Total sample *n* = 59	Low/median completion subgroup *n* = 19	Good/ excellent completion subgroup *n* = 40	*P*-value
Variables				
Age in years, mean (SD)	38.7 (11.2)	33.6 (10.4)	41.1 (10.8)	*P* = 0.02^a^
Gender				*P* = 0.13^b^
Female, n (%)	52 (88.1)	15 (78.9)	37 (92.5)	
Male, n (%)	7 (11.9)	4 (21.1)	3 (7.5)	
Years of work experience, mean (SD)	15.8 (11.8)	10.6 (10.8)	18.3 (11.5)	*P* = 0.02^a^
Employment status				*P* = 0.01^b^
Part-time (<100 %), n (%)	27 (45.8)	4 (21.1)	23 (57.5)	
Full-time (100 %), n (%)	32 (54.2)	15 (78.9)	17 (42.5)	
Type of shift work				*P* = 0.49^b^
Day shift, n (%)	58 (98.3)	19 (100)	39 (97.5)	
Night shift, n (%)	1 (1.7)	0 (0)	1 (2.5)	
Educational level				*P* = 0.18^b^
Certificate degree, n (%)	10 (17.0)	1 (5.2)	9 (22.5)	
Bachelor degree, n (%)	41 (69.5)	14 (73.7)	27 (67.5)	
Master degree, n (%)	8 (13.5)	4 (21.1)	4 (10.0)	
Delirium training last 5 years				*P* = 0.73^b^
Yes, n (%)	8 (13.5)	3 (15.8)	5 (12.5)	
No, n (%)	51 (86.5)	16 (84.2)	35 (87.5)	

*Abbreviations:* SD = standard deviation

^a^ANOVA test

^b^Chi-square test

### Completion of the e-learning tool

The low/moderate completion (L/MC, for definition see analysis section) subgroup included 19 (32.2 %) participants, of whom 2 did not start the e-learning tool. The good/excellent completion (G/EC) subgroup included 40 (67.8 %) participants. Almost half of the healthcare workers (*n* = 26; 44.1 %) finalized all the modules. For those who started using the e-learning tool, the mean number of completed modules per participant was 8.2 (SD 3.2). The mean time to complete the modules for those in the low/moderate completion subgroup was 31.8 min (SD 60.8) and 115.6 min (SD 54.6) for those in the good/excellent completion subgroup, respectively. There were no statistically significant differences in demographic data between the two completing groups, except for age, employment status and number of years of work experience (Table 3).

### Effect of the e-learning tool on outcomes

#### Delirium recognition (DR)

More healthcare workers in the posttest phase were able to correctly identify hypoactive (64.9 % versus (vs.) 45.6 %; *P* = 0.04) and hyperactive (93.0 % vs. 71.9 %; *P* = 0.007) delirium compared to the pretest phase, respectively. The mean total DR score also significantly improved (3.1 ± 0.9 vs 2.7 ± 1.1; *P* = 0.04, Cohen’s *d* = 0.38) (Table 4).

**Table 4:** Healthcare Workers’ Delirium Recognition, Their Knowledge about Delirium and Strain in Caring for Delirious Patients in the Pretest and Posttest Phase (*n* = 57^a^)

Variable	Pretest phase (*n* = 57)	Posttest phase (*n* = 57)	*P*-value
Delirium recognition – ability to identify delirium			
*Cases, n correct (%)*			
Dementia	41 (71.9)	44 (77.2)	0.55^b^
Hypoactive delirium	26 (45.6)	37 (64.9)	0.04^b^
Hyperactive delirium	41 (71.9)	53 (93.0)	0.007^b^
Dementia + hyper-/hypoactive delirium	49 (86.0)	45 (78.9)	0.31^b^
Total DR score, mean (SD) (range 0–4)	2.7 (1.1)	3.1 (0.9)	0.04^c^
Knowledge about delirium			
Total DKQ score, mean (SD) (range 0–35)	28.3 (4.5)	31.7 (2.6)	<0.001^c^
Strain in caring for delirious patients			
Total SCDI score, mean (SD) (range 20–80)	50.9 (9.2)	51.2 (8.4)	0.46^c^
Subscore hypoactive behavior, mean (SD) (range 3–12)	7.3 (1.8)	6.9 (1.7)	0.29^c^
Subscore hypoalert behavior, mean (SD) (range 4–16)	8.9 (2.1)	8.8 (1.7)	0.84^c^
Subscore fluctuating course/psychoneurotic behavior,			
mean (SD) (range 5–20)	11.2 (2.9)	11.3 (3.0)	0.51^c^
Subscore hyperactive/hyperalert behavior, mean (SD)	23.7 (4.2)	23.9 (4.2)	0.71^c^
(range 8–32)			

*Abbreviations: SD* standard deviation, *DR* delirium recognition, *DKQ* Delirium Knowledge Questionnaire, *SCDI* Strain of Care for Delirium Index

^a^This type of analysis included only the healthcare workers who completed minimum 1 module of the delirium e-learning tool

^b^McNemar test

^c^Paired *t*-test

The unadjusted linear mixed model noted a statistically significant difference in the change of mean total DR scores over time between the L/MC subgroup and the G/EC subgroup (*P* = 0.03), with a difference estimate (DE) of 0.81 (95 % CI 0.05–1.57). The difference in the change of mean total DR scores over time between the two subgroups was no longer significant in the adjusted linear mixed model (DE: 0.76; 95 % CI −0.06–1.6; *P* = 0.07). The unadjusted model showed a significant increase of the mean total DR score in the posttest within the G/EC subgroup compared to the pretest phase (DE: 0.6; 95 % CI 0.17–1.03; *P* = 0.007). After controlling for potential confounders, no change in the mean total DR scores within this subgroup was noted (adjusted DE: 0.49; 95 % CI −0.26–1.24; *P* = 0.19). Both in the unadjusted and adjusted models, the other group comparison of changes over time were not statistically significant.

A weak, but significant correlation between the number of completed e-learning modules and the change scores of the total DR scores was found (*r*_*P*_ = 0.3; *P* = 0.02).

#### Knowledge about delirium

The proportion of correct answers on all the DKQ items was higher in the posttest phase compared to the pretest phase, except for 7 items (items 15, 18, 20, 23, 24, 29, 34) (Table 2). Moreover, in 16 items, the difference in proportion of correct answers was minimum 10 % in favor of the posttest phase. Only item 18 was answered more correctly in the pretest. The mean total DKQ score of healthcare workers in the posttest phase was statistically significant improved compared to the pretest phase (31.7 ± 2.6 vs. 28.3 ± 4.5; *P* < 0.001, Cohen’s *d* = 0.76).

Both the unadjusted and adjusted linear mixed models showed no statistically significant difference in change of mean total DKQ scores over time between the L/MC subgroup and the G/EC subgroup (unadjusted DE: 1.5; 95 % CI −0.59- 3.55; *P* = 0.16 versus (vs) adjusted DE: 0.95; 95 % CI −1.26 – 3.16; *P* = 0.39). Nevertheless, within the G/EC subgroup there was a significant increase of mean total DKQ scores in the posttest compared to the pretest phase (unadjusted DE: 3.4; 95 % CI 2.20–4.55; *P* < 0.001 vs adjusted DE: 2.4; 95 % CI 0.36 – 4.40; *P* = 0.02). Within the L/MC subgroup, the mean total DKQ scores in the posttest phase were also significantly increased (unadjusted DE: 1.89; 95 % CI 0.18–3.60; *P* = 0.03), but significance disappeared in the adjusted model (DE: 1.4; 95 % CI–0.77 - 3.61; *P* = 0.19).

There was a weak, albeit significant correlation between the number of completed e-learning modules and the change scores of the total DKQ scores (rho = 0.3; *P* = 0.04).

#### Strain in caring for delirious patients

There were no significant differences between the posttest and pretest phase in mean total SCDI scores (*P* = 0.46) and its 4 mean subscale scores (Table 4).

Also unadjusted and adjusted linear mixed model analysis revealed no statistically significant difference in change of mean total SCDI scores over time between the L/MC subgroup and the G/EC subgroup (unadjusted DE: −0.07; 95 % CI −3.33 – 3.18; *P* = 0.96 vs adjusted DE: 0.43; 95 % CI −3.05 - 3.91; *P* = 0.81). There was no significant difference in the mean total SCDI score in the posttest compared to the pretest phase within the L/MC subgroup (unadjusted DE: 0.47; *P* = 0.7 vs adjusted DE: −0.61; *P* = 0.72) and within the G/EC subgroup (unadjusted DE: 0.4; *P* = 0.67 vs adjusted DE: −0.18; *P* = 0.91).

No correlations between the number of completed e-learning modules and neither the total nor subscale SCDI change scores were detected (data not shown/available upon request from the authors).

## Discussion

This is the first study investigating the effect of a delirium e-learning tool consisting of 11 modules on healthcare workers’ delirium recognition, knowledge and level of delirium strain, taking into account the amount of completed modules. Consistent with previous research [16–18, 25], our findings support that e-learning might be an effective tool for improving healthcare workers’ knowledge and recognition of delirium. Moreover, the difference in total delirium knowledge scores before and after using the e-learning tool was found to be moderate and although the difference in total delirium detection levels was rather small, the e-learning tool led to a 20 % to 21 % higher proportion of correctly identified hypoactive and hyperactive delirium cases, respectively. Because of the well-known under recognition of delirium in clinical practice [4, 5], those differences were not only statistically significant but also highly clinically relevant.

Although our study findings are in line with previous results indicating positive effects of e-learning on nurses’ delirium recognition [18] and knowledge [17, 18], comparability of the studies is limited because of different study designs, analysis and measurement instruments. Our study expands the existing knowledge on delirium e-learning [17, 18], as it evaluated the effect of e-learning on healthcare workers’ delirium strain, and investigated its effect on their recognition and knowledge about delirium by taking into account the amount of completed modules. Moreover, our findings suggest that the effect of the e-learning tool on delirium recognition and knowledge is causally related to its level of completion, highlighting the importance of motivating healthcare workers to complete the full e-learning tool. This was demonstrated by a significant association between the number of completed modules and the level of DR change scores, as well as by a significant difference in the change of DR levels over time between healthcare workers who completed 0 to 6 modules and those who completed 7 to 11 modules, in which the improvement was only statistically significant within the latter group. After controlling for potential confounding factors, the difference in the change of DR levels over time between healthcare workers in the 0–6 module subgroup and those in the 7–11 module subgroup was no longer significant. Yet, there was a trend towards borderline significance. Furthermore, there was a small but significant association between the number of completed modules and the level of DKQ change scores. Although - independent of the controlling for potential confounders - the difference in the change of DKQ levels over time between the two subgroups was not significant, the DKQ scores were significantly improved in the posttest phase compared to the pretest phase in healthcare workers who completed 7–11 modules. On the other hand, our study showed no effect of the e-learning tool on healthcare workers’ strain whether or not taking into account the amount of completed modules. However, although previous research in delirium [26] and dementia [27, 28] care provided evidence that knowledge in combination with other factors, such as perceived caring climate of the ward, the possibilities to reflect about practice, staff age, emotional and management support, and communication difficulties with patients, are factors related with experienced care strain, additional studies are needed to investigate the predictors of delirium care strain and its relation to delirium education through e-learning.

Our e-learning tool holds promise in improving delirium detection and knowledge because of its flexibility regarding the time of training, its ability to standardize teaching materials, its potential to implement efficiently to large groups and its relatively low cost (development cost only). For these reasons [11, 14], e-learning has been suggested as an alternative learning method especially in busy healthcare workers. Nevertheless, a previous feasibility study revealed that the lack of interactivity and the need to have sufficient self-discipline to complete the tool without supervision were barriers to e-learning [20]. Therefore, alternative forms of e-learning should be explored. It might be necessary to use the tool in combination with a delivery schedule over fixed time periods and recurrent feedback sessions organized by a facilitator. Structuring e-learning in such a format has been shown to hold promise in medical education [13]. Furthermore, to reach real changes in delirium care in practice, e-learning needs to be seen as one component within a larger approach of interprofessional blended-learning education extended with enabling and reinforcing strategies including restructuring of practice [29, 30].

Some methodological limitations need to be considered. First, a pretest/posttest design was used, and further testing using a randomized controlled trial (RCT) design is warranted. However, RCT’s are notoriously hard to conduct in education research because education is a social process and heavily influenced by contextual factors which cannot be controlled against. Therefore a large scale clustered RCT with multiple sites would be required and even then may not do the intervention justice. Second, because the study was conducted in a voluntary sample of healthcare workers, this sample might include only the most motivated people which might have induced bias and limits its generalizability. Third, quantitative data indicated the time pressure during working hours as an important reason for not completing all the modules. However, an in-depth qualitative interview might have been given more valuable information to identify why there was such a high attrition rate. Fourth, the level of knowledge of the sample in our hospital was already relatively high, which might affect transferability of the effect to other settings. Nonetheless, a change in delirium recognition and knowledge were observed. Fifth, the knowledge about delirium was assessed with the DKQ, an instrument developed for this study that supports good content and face validity based on expert review and pilot testing. However, additional validity and reliability testing is needed. Sixth, since the effects of the e-learning tool on delirium recognition, knowledge and strain in caring for delirious patients were evaluated once after a 2-month learning period, no statements about the long term effects could be made and as a consequence future studies should also focus on the long term effects.

Despite these caveats, this study has several important strengths, including the use of validated instruments to assess healthcare workers’ levels of subjective strain and delirium recognition, the detailed statistical analyses taking account of different parameters, the organization of the self-directed e-learning tool into 11 modules in which theory is combined with videos, case-studies and tests for self-assessment, its development via a robust process and feasibility testing, and the tracking of compliance with the e-learning tool.

## Conclusion

In general, the on-line delirium education as delivered by the e-learning tool improved healthcare workers’ delirium recognition and knowledge, but had no effect on their level of strain. The effect of this tool on healthcare workers’ delirium recognition and knowledge was related to its level of completion. However, this relation was less explicit after controlling for potential confounders warranting further investigation. Nonetheless, the study findings are particularly important as potentially large numbers of healthcare workers can be trained with a relatively inexpensive tool (development cost only). Since studies have shown the impact of educational approaches on the prevention of delirium, an e-learning tool, such as ours, could potentially reduce the incidence of delirium in clinical practice. Larger scale studies are warranted to replicate our promising findings.

## Abbreviations

DSD: Delirium superimposed on dementia
DR: Delirium recognition
DKQ: Delirium Knowledge Questionnaire
SCDI: Strain of Care for Delirium Index
N/LC: No/low completion
MC: Moderate completion
G/EC: Good/excellent completion
SD: Standard deviation
DE: Difference estimate

## References

[1] American Psychiatric Association (2013). Neurocognitive disorders. Diagnostic and statistical manual of mental disorders.

[2] Siddiqi N, Holmes JD, House AO (2006). Occurrence and outcome of delirium in medical in-patients: a systematic literature review. Age Ageing.

[3] Young J, Murthy L, Westby M, Akunne A, O’Mahony R (2010). Diagnosis, prevention, and management of delirium: summary of NICE guidance. Brit Med J.

[4] Inouye S, Foreman M, Mion L, Katz KH, Cooney LM (2001). Nurses’ recognition of delirium and its symptoms: comparison of nurse and researcher ratings. Arch Intern Med.

[5] Steis MR, Fick DM (2008). Are nurses recognizing delirium? A systematic review. J Gerontol Nurs.

[6] Teodorczuk A, Mukaetova-Ladinska E, Corbett S, Welfare M (2013). Reconceptualising models of delirium education: Findings of a Grounded Theory study. Int Psychogeriatr.

[7] Witlox J, Eurelings LSM, de Jonghe JFM, Kalisvaart KJ, Eikelenboom P, van Gool WA (2010). Delirium in elderly patients and the risk of postdischarge mortality, institutionalization, and dementia. J Am Med Assoc.

[8] Wand APF (2011). Evaluating the effectiveness of educational interventions to prevent delirium. Aust J Ageing.

[9] Yanamadala M, Wieland D, Heflin MT (2013). Educational interventions to improve recognition of delirium: a systematic review. J Am Geriatr Soc.

[10] Pretto M, Milisen K, Spirig R, De Geest S, Regazzoni P, Hasemann W (2009). Effects of an interdisciplinary nurse-led Delirium Prevention and Management Program (DPMP) on nursing workload: A pilot study. Int J Nurs Stud.

[11] Irving K, Detroyer E, Foreman M, Milisen K (2009). The virtual gateway: opening doors in delirium teaching and learning. Int Rev Psychiatry.

[12] Teodorczuk A, Reynish E, Milisen K (2012). Improving recognition of delirium in clinical practice: A Call for Action. BMC Geriatr.

[13] Curran VR, Fleet LJ, Kirby F (2010). A comparative evaluation of the effect of internet-based CME delivery format on satisfaction, knowledge and confidence. BMC Med Educ.

[14] Walsh K, Rutherford A, Richardson J, Moore P (2010). NICE medical education modules: an analysis of cost-effectiveness. Educ Prim Care.

[15] Joint Information Systems Committee: E-learning. www.jisc.ac.uk/elearning (2013). Accessed 23 Oct 2013.

[16] Cook DA, Levinson AJ, Garside S, Dupras DM, Erwin PJ, Montori VM (2008). Internet-based learning in the health professions. J Am Med Assoc.

[17] van de Steeg L, Ijkema R, Langelaan M, Wagner C (2014). Can an e-learning course improve nursing care for older people at risk of delirum: a stepped wedge cluster randomised trial. BMC Geriatr.

[18] McCrow J, Sullivan KA, Beattie ER (2014). Delirium knowledge and recognition: a randomized controlled trial of web-based educational intervention for acute care nurses. Nurse Educ Today.

[19] Mc Donnell S, Timmins F (2012). A quantitative exploration of the subjective burden experienced by nurses when caring for patients with delirium. J Clin Nurs.

[20] Detroyer E, Joosten E, Milisen K (2014). An interactive e-learning tool about delirium for healthcare providers: development and testing of feasibility. Ann Delirium Care.

[21] Fick DM, Hodo DM, Lawrence F, Inouye SK (2007). Recognizing delirium superimposed on dementia: Assessing nurses’ knowledge using case vignettes. J Gerontol Nurs.

[22] Hare M, Wynaden D, McGowan S (2008). A questionnaire to determine nurses’ knowledge of delirium and its risk factors. Contemp Nurse.

[23] Milisen K, Cremers S, Foreman MD, Vandevelde E, Haspeslagh M, De Geest S (2004). The strain of care for Delirium Index: a new instrument to assess nurses’ strain in caring for patients with delirium. Int J Nurs Stud.

[24] Cohen J (1988). Statistical power analysis for the behavioral sciences.

[25] Chao SK, Brett B, Wiecha JM, Norton LE, Levine SA (2012). Use of an online curriculum to teach delirium to fourth-year medical students: a comparison with lecture format. J Am Geriatric Soc.

[26] Hallberg IR (1999). Impact of delirium on professionals. Dement Geriatr Cogn Disord.

[27] Edberg AK, Bird M, Richards DA, Woods R, Keeley P, Davis-Quarrell V (2008). Strain in nursing care of people with dementia: Nurses’ experience in Australia, Sweden and United Kingdom. Aging Ment Health.

[28] Evardsson D, Sandman PO, Nay R, Karlsson S (2009). predictors of job strain in residential dementia care nursing staff. J Nurs Manag.

[29] Sockalingam S, Tan A, Hawa R, Pollex H, Abbey S, Hodges BD (2014). Interprofessional education for delirium care: a systematic review. J Interprof Care.

[30] Teodorczuk A, Mukaetova-Ladinska E (2014). Delirium interventions should address negative attitudes and focus on promoting learning through practice. J Am Geriatr Soc.

